# [Retracted] A disintegrin and metalloprotease 17 promotes microglial cell survival via epidermal growth factor receptor signalling following spinal cord injury

**DOI:** 10.3892/mmr.2024.13414

**Published:** 2024-12-09

**Authors:** Zijian Wei, Deshui Yu, Yunlong Bi, Yang Cao

Mol Med Rep 12: 63–70, 2015; DOI: 10.3892/mmr.2015.3395

Following the publication of this paper, it was drawn to the Editor's attention by a concerned reader that certain of the cell apoptotic data shown in Fig. 3A, the flow cytometric (FCM) data in Fig. 3B on p. 67, and the western blot data shown in Fig. 5 on p. 68 were strikingly similar to data that had either already been submitted for publication elsewhere, or which subsequently appeared in different form in other articles/publications. Moreover, patterns of data featured within certain quadrants of the FCM plots featured in Fig. 5 appeared to be strikingly similiar to other patterns of data when comparing between the quadrants of the FCM plots within this same figure, such that the similarities were difficult to attribute to coincidence.

Owing to the fact that the abovementioned data have apparently subsequently appeared in other unrelated articles, and owing to the potentially anomalous presentation of data in the FCM plots in Fig. 5, the Editor of *Molecular Medicine Reports* has decided that this paper should be retracted from the Journal on the grounds of an overall lack of confidence in the presented data. The authors were asked for an explanation to account for these concerns, but the Editorial Office did not receive a reply. The Editor apologizes to the readership for any inconvenience caused.

